# Supplementary material for: Selective gene expression maintains human tRNA anticodon pools during differentiation
Source: Nat Cell Biol. 2024 Jan 8;26(1):100–12. doi: 10.1038/s41556-023-01317-3 (PMC10791582; doi:10.1038/s41556-023-01317-3)
Supplement: Supplementary file 1 — Reporting Summary [file 41556_2023_1317_MOESM1_ESM.pdf]

Corresponding author(s): Danny D. Nedialkova

Last updated by author(s): Oct 22, 2023

Reporting Summary

Nature Portfolio wishes to improve the reproducibility of the work that we publish. This form provides structure for consistency and transparency in reporting. For further information on Nature Portfolio policies, see our Editorial Policies and the Editorial Policy Checklist.

Statistics

For all statistical analyses, confirm that the following items are present in the figure legend, table legend, main text, or Methods section.

n/a	Confirmed	
☐	☒	The exact sample size (*n*) for each experimental group/condition, given as a discrete number and unit of measurement
☐	☒	A statement on whether measurements were taken from distinct samples or whether the same sample was measured repeatedly
☐	☒	The statistical test(s) used AND whether they are one- or two-sided<br>*Only common tests should be described solely by name; describe more complex techniques in the Methods section.*
☐	☒	A description of all covariates tested
☐	☒	A description of any assumptions or corrections, such as tests of normality and adjustment for multiple comparisons
☐	☒	A full description of the statistical parameters including central tendency (e.g. means) or other basic estimates (e.g. regression coefficient) AND variation (e.g. standard deviation) or associated estimates of uncertainty (e.g. confidence intervals)
☐	☒	For null hypothesis testing, the test statistic (e.g. *F*, *t*, *r*) with confidence intervals, effect sizes, degrees of freedom and *P* value noted<br>*Give P values as exact values whenever suitable.*
☒	☐	For Bayesian analysis, information on the choice of priors and Markov chain Monte Carlo settings
☒	☐	For hierarchical and complex designs, identification of the appropriate level for tests and full reporting of outcomes
☐	☒	Estimates of effect sizes (e.g. Cohen's *d*, Pearson's *r*), indicating how they were calculated

Our web collection on statistics for biologists contains articles on many of the points above.

Software and code

Policy information about availability of computer code

Data collection

Western blot images were collected with iBiright Analysis Software (Thermo Fisher). Norther blot images were collected on a Typhoon FLA 9000 (GE Healthcare).

Data analysis

R v4.2.2 and Python v3.7 were used for analysis of NGS data, in addition to the following command-line software:
mimseq v1.2 (https://github.com/nedialkova-lab/mim-tRNAseq/tree/master/mimseq)
Customized scikit-ribo v0.2.4b1 for use on human genome (https://github.com/nedialkova-lab/scikit-ribo-ext).
STAR v2.6.1.c
cutadapt v3.5
Trim Galore v0.6.4
RSEM v1.3.1
Kallisto v0.44.0
Picard Tools MarkDuplicates v2.17.10
mmquant v1.3
deepTools alignmentSieve v3.4.0
deepTools v3.5.1
MACS v2.2.6
bedtools v2.29.2
Infernal v1.1.2
Phantompeakqualtools v1.2.2
tRNet CNN model (https://github.com/nedialkova-lab/tRNet)

R packages:
DESEq2 v1.38.1
edgeR v3.34.1
ComplexHeatmap v2.14.0
DiffBind v3.2.7
ChIPpeakAnno v3.26.4
universalmotif v1.16.0
phantompeakqualtools v1.2.2

Python packages:
logomaker v0.8
keras v2.2.4
SHAP v0.29.3
Tensorflow v1.15.5
TF-Modisco v0.5.14.1

Online tools:
MEME v5.5.4 (https://meme-suite.org/meme/tools/meme)

For manuscripts utilizing custom algorithms or software that are central to the research but not yet described in published literature, software must be made available to editors and reviewers. We strongly encourage code deposition in a community repository (e.g. GitHub). See the Nature Portfolio guidelines for submitting code & software for further information.

Data

Policy information about availability of data

All manuscripts must include a data availability statement. This statement should provide the following information, where applicable:

- Accession codes, unique identifiers, or web links for publicly available datasets
- A description of any restrictions on data availability
- For clinical datasets or third party data, please ensure that the statement adheres to our policy

High-throughput sequencing data has been deposited in the Gene Expression Omnibus Database (GSE227928). Public genome-wide bisulfite sequencing data used here is available through ENCODE project ENCSR617FKV (GEO: GSE80911). Source data have been provided in Source Data. All other data supporting the findings of this study are available from the corresponding author on reasonable request.

Research involving human participants, their data, or biological material

Policy information about studies with human participants or human data. See also policy information about sex, gender (identity/presentation), and sexual orientation and race, ethnicity and racism.

Reporting on sex and gender	N/A
Reporting on race, ethnicity, or other socially relevant groupings	N/A
Population characteristics	N/A
Recruitment	N/A
Ethics oversight	N/A

Note that full information on the approval of the study protocol must also be provided in the manuscript.

Field-specific reporting

Please select the one below that is the best fit for your research. If you are not sure, read the appropriate sections before making your selection.

☒ Life sciences ☐ Behavioural & social sciences ☐ Ecological, evolutionary & environmental sciences

For a reference copy of the document with all sections, see nature.com/documents/nr-reporting-summary-flat.pdf

Life sciences study design

All studies must disclose on these points even when the disclosure is negative.

Sample size	No statistical method was used to determine appropriate sample sizes. For all sequencing datasets where comparative statistical analysis was performed, a sample size of two was chosen to allow such statistical tests at an affordable cost. Sample sizes are indicated in the figure legends.

Data exclusions	No data was excluded.
Replication	For tRNA-seq, ATAC-seq, RNA-seq and ChIP-seq experiments, 2 biological replicates (independent differentiations) were performed, and correlation analysis were conducted to ensure the consistency between replicates. For H3K27me3 ChIP-seq, H3K9me3 ChIP-Seq, and H3K4me3 ChIP-Seq in NPC, a single replicate per cell type were performed. For ribosome profiling where cycloheximide (CHX) and tigecycline (TIG) were present in lysis buffer, 2 biological replicates for hiPSC and NPC were performed, while only one replicate was performed for the CHX-only sample. For CRISPRi and following functional studies, 2 clones were selected for each sgRNA. All attempts of replications were successful.
Randomization	No randomization was performed. This study was carried out in the kucg_2 hiPSC line and its differentiated counterparts, as well as in wibj_2 hiPSC cells and HEK293T/17. Covariates control is not applicable due to the small number of cell lines used.
Blinding	The investigators were not blinded to the group as no human subjects or clinical samples were involved and no subjective measurements were taken.

Reporting for specific materials, systems and methods

We require information from authors about some types of materials, experimental systems and methods used in many studies. Here, indicate whether each material, system or method listed is relevant to your study. If you are not sure if a list item applies to your research, read the appropriate section before selecting a response.

Materials & experimental systems

n/a	Involved in the study
☐	☒ Antibodies
☐	☒ Eukaryotic cell lines
☒	☐ Palaeontology and archaeology
☒	☐ Animals and other organisms
☒	☐ Clinical data
☒	☐ Dual use research of concern
☒	☐ Plants

Methods

n/a	Involved in the study
☐	☒ ChIP-seq
☒	☐ Flow cytometry
☒	☐ MRI-based neuroimaging

Antibodies

Antibodies used	Anti-POU5F1 C-10 (1:400; Santa Cruz, #sc-5279)<br>Anti-SOX2 E-4 (1:200; Santa Cruz, #sc-365823)<br>Anti-NANOG P1-2D8 (1:200; DSHB Hybridoma Product PCRP-NANOGP1-2D8)<br>Anti-PAX6 (1:200; Abcam #ab5790)<br>Anti-Nestin (1:200; R&D Systems, #MAB1259)<br>Anti-MAP2 (1:1000; Abcam, #ab92434)<br>Anti-CHAT (1:200; Abcam, #ab6168)<br>Anti-cTNT (1:5; CT3, deposited to the DSHB by Lin, J.J-C.)<br>Anti-ACTN2 (1:800; Sigma-Aldrich #A7811)<br>Goat anti-mouse Alexa Fluor 488 (1:2000; Thermo Fisher Scientific, #A-11001)<br>Goat anti-rabbit Alexa Fluor 488 (1:2000; Thermo Fisher Scientific, #A-11034)<br>Goat anti-mouse Alexa Fluor 633 (1:500; Thermo Fisher Scientific, #A-21052)<br>Anti-POLR3A/RPC1 (1:1000 for immunoblotting and 5 µg for ChIP; Cell Signaling Technology, #12825)<br>Drosophila spike-in antibody (0.2 µg for ChIP; Active Motif, #61686)<br>Anti-H3K4me3 (1:200; Active Motif, #39159)<br>Anti-H3K27me3 (1:200; Millipore, #07-449)<br>Anti-H3K9me3 (1:200; Cell Signaling, #13969)<br>Anti-POLR3G/RPC7α (1:1000 for immunoblotting and 1:100 for ChIP; Santa Cruz, #sc21754)<br>Anti-POLR3B/RPC2 (1:1000; Santa Cruz; #sc-515362)<br>Anti-BRF1 (1:100; Abcam, #ab264191)<br>Anti-MAF1 (1:1000; Santa Cruz; #sc-515614 X)<br>Anti-phospho-4E-BP1 (1:1000; Cell Signaling, #2855T)<br>Anti-phospho-p70 S6 Kinase (T389) (1:1000; Cell Signaling, #9206S)<br>Anti-4E-BP1 (1:1000; Cell Signaling, #9644)<br>Anti-p70 S6 Kinase (1:1000; Cell Signaling, #2708T)<br>Anti-vinculin (1:1000; Cell Signaling; #13901)<br>Anti-mouse IgG-HRP, 1:4000; Dianova, #115-035-003<br>Anti-rabbit IgG-HRP (1:4000; Dianova, #111-035-003)
Validation	Anti-POU5F1 C-10 (Santa Cruz, #sc-5279): according to the manufacturer, this mouse monoclonal antibody is raised against amino acids 1-134 of Oct-3/4 of human origin recommended for is recommended for detection of Oct-3/4 of mouse, rat and human origin by immunofluorescence; non cross-reactive with Oct-3/4 isoform B; cited in >2450 publications (https://www.scbt.com/p/oct-3-4-

antibody-c-10?productCanUrl=oct-3-4-antibody-c-10&_requestid=526329)

Anti-SOX2 E-4 (Santa Cruz, #sc-365823): according to the manufacturer, this mouse monoclonal antibody is specific for an epitope mapping between amino acids 170-201 within an internal region of Sox-2 of human origin and is recommended for is recommended for detection of Sox-2 of mouse, rat and human origin by immunofluorescence; cited in >260 publications (https://www.scbt.com/p/sox-2-antibody-e-4?requestFrom=search).

Anti-NANOG P1-2D8 (DSHB Hybridoma Product PCRP-NANOGP1-2D8): according to the manufacturer, this monoclonal antibody was raised against amino acids 1-127 of the human NANOG protein and is recommended for detecting human NANOG by immunofluorescence (https://dshb.biology.uiowa.edu/PCRP-NANOGP1-2D8).

Anti-PAX6 (Abcam #ab5790): according to the manufactirer, this polyclonal antibody is suitable for detecting PAX6 from mouse, human, rat, and monkey by immunofluorescence; cited in >100 publications (https://www.abcam.com/products/primary-antibodies/pax6-antibody-ab5790.html?productWallTab=ShowAll)

AAnti-Nestin (R&D Systems, #MAB1259): according to the manufactirer, this monoclonal antibody detects human nestin by immunofluorescence; cited in >100 publications (https://www.rndsystems.com/products/human-nestin-antibody-196908_mab1259?gclid=Cj0KCQjw4NujBhC5ARIsAF4Iv6eJ-nyTOhCazimeegmTVBXGL9pW6doy4o12apC7F5i93ffSmtPhOxUaAtgNEALw_wcB&gclsrc=aw.ds#product-details)

Anti-MAP2 (Abcam, #ab92434): according to the manufactirer, this polyclonal antibody detects human MAP2 by immunofluorescence; cited in >45 publications (https://www.abcam.com/products/primary-antibodies/map2-antibody-ab92434.html)

Anti-CHAT (Abcam, #ab6168): according to the manufactirer, this polyclonal antibody was raised against a peptide corresponding to amino acids 168-189 of Choline Acetyltransferase and reacts with the protein in immunohistochemitry; cited in >13 publications (https://www.abcam.com/products/primary-antibodies/choline-acetyltransferase-antibody-ab6168.html)

Anti-cTNT (CT3, deposited to the DSHB by Lin, J.J-C.): initially published in initially published in: Jin, J., Lin, J.-C., and Lin, J.J.-C. (1990). Troponin T isoform switching during heart development. Ann. NY Acad. Sci. 588, 393-396. CT3 recognizes the embryonic and adult cardiac isforms [PMID: 2358124]. CT3 cross-react with slow skeletal muscle TnT but doesn't recognize fast skeletal muscle TnT [PMID: 12732643]. Cited in >99 publications (https://dshb.biology.uiowa.edu/CT3)

Anti-ACTN2 (Sigma-Aldrich #A7811): according to the manufacturer, this monoclonal antibody is suitable for detecting human α-Actinin by immunofluorescence; cited in >970 publications (https://www.sigmaaldrich.com/DE/en/product/sigma/a7811).

Anti-POLR3A/RPC1 (Cell Signaling Technology, #12825): according to the manufacturer, this monoclonal antibody is produced by immunizing animals with a synthetic peptide corresponding to residues surrounding Val613 of human POLR3A protein and has been validated for use in ChIP-Seq (https://www.cellsignal.com/products/primary-antibodies/polr3a-d5y2d-rabbit-mab/12825). Additional validation we performed included Western blotting (single band at the expected MW of POLR3A/RPC1) and extensive characterization of ChIP-Seq datasets (presence of clearly defined strong peaks at Pol III target genes and absence of ChIP signal from other genomic regions).

Drosophila spike-in antibody (Active Motif, #61686): according to the manufacturer, the spike-in antibody recognizes a histone variant that is specific to the species of the Spike-in Chromatin (Drosophila); cited in >25 publications (https://www.activemotif.com/catalog/1091/chip-normalization)

Anti-H3K4me3 (Active Motif, #39159): according to the manufacturer, this Histone H3 trimethyl Lys4 (H3K4me3) polyclonal antibody was raised against a peptide including trimethyl-lysine 4 of histone H3 and its specificity was confirmed by dot bot analysis (https://www.activemotif.com/catalog/details/39159/histone-h3-trimethyl-lys4-antibody-pab); it has been validated for ChIP by modENCODE (https://compbio.med.harvard.edu/antibodies/antibodies/84).

Anti-H3K27me3 (Millipore, #07-449): according to the manufacturer, this polyclonal antibody is dot blot tested for trimethylated lysine 27 specificity and validated in immunoprecipitation (https://www.merckmillipore.com/DE/de/product/Anti-trimethyl-Histone-H3-Lys27-Antibody,MM_NF-07-449?ReferrerURL=https%3A%2F%2Fwww.google.com%2F#); it has been validated for ChIP-Seq by modENCODE (https://compbio.med.harvard.edu/antibodies/antibodies/57).

Anti-H3K9me3 (Cell Signaling, #13969): according to the manufacturer, this monoclonal antibody detects endogenous levels of histone H3 when tri-methylated on Lys9. It shows some cross-reactivity with histone H3 that is di-methylated on Lys9, but does not cross-react with non-methylated or mono-methylated histone H3 Lys9. This antibody does not detect tri-methyl histone H3 Lys9 when the adjacent Ser10 residue is phosphorylated during mitosis. In addition, this antibody does not cross-react with methylated histone H3 Lys4, Lys27, Lys36, or Lys79. This antibody has been validated using SimpleChIP® Enzymatic Chromatin IP Kits. (https://www.cellsignal.com/products/primary-antibodies/tri-methyl-histone-h3-lys9-d4w1u-rabbit-mab/13969); cited in >112 publications.

Anti-POLR3G/RPC7α (Santa Cruz, #sc21754): according to the manufacturer, this monoclonal antibody raised against recombinant human RPC32/POLR3G/RPC7α subunit of RNA polymerase III and cited in >6 publications (https://www.scbt.com/p/pol-iii-rpc32-antibody-c32-1). Additional validation we performed included Western blotting (single band at the expected MW of POLR3G and its substantial decrease upon POLR3G knockdown by CRISPRi) and extensive characterization of ChIP-Seq datasets (presence of clearly defined strong peaks at Pol III target genes and absence of ChIP signal from other genomic regions).

Anti-BRF1 (Abcam, #ab264191): according to the manufacturer, this polyclonal antibody was raised against a synthetic peptide within human BRF1 aa 627-677 (https://www.abcam.com/products/primary-antibodies/brf1-antibody-ab264191.html). Additional validation we performed included Western blotting (single band at the expected MW of BRF1 and its substantial decrease upon BRF1 knockdown by CRISPRi) and extensive characterization of ChIP-Seq datasets (presence of clearly defined strong peaks at Pol III target genes and absence of ChIP signal from other genomic regions, inlcuding RNU6-1, at which Pol III is assembled via BRF2).

Anti-MAF1 (Santa Cruz; #sc-515614 X): according to the manufacturer, this monolconal antibody specific for an epitope mapping between amino acids 99-122 within an internal region of MAF1 of human origin; cited in 3 publications (https://www.scbt.com/p/maf1-antibody-h-2). Additional validation we performed included Western blotting (single band or smear at the expected MW of MAF1 depending on phosphorylation status and its substantial decrease upon MAF1 knockdown by CRISPRi).

Anti-phospho-4E-BP1 (Cell Signaling, #2855T): according to the manufacturer, this monoclonal antibody detects endogenous levels of 4E-BP1 only when phosphorylated at Thr37 and/or Thr46. This antibody may cross-react with 4E-BP2 and 4E-BP3 when phosphorylated at equivalent sites; cited in >1680 publications (https://www.cellsignal.com/products/primary-antibodies/phospho-4e-bp1-thr37-46-236b4-rabbit-mab/2855). Additional validation we performed included Western blotting (several bands at the expected MW of phoshorylated 4E-BP1 and the disappearance of a subset of those upon mTORC1 inhibition by Torin 1 treatment).

Anti-phospho-p70 S6 Kinase (T389) (Cell Signaling, #9206S): according to the manufacturer, this monoclonal antibody detects endogenous levels of p70 S6 kinase only when phosphorylated at Thr389. This antibody also detects p85 S6 kinase when phosphorylated at the analogous site (Thr412) and possibly S6KII phosphorylated at Thr388; cited in >530 publications (https://www.cellsignal.com/products/primary-antibodies/phospho-p70-s6-kinase-thr389-1a5-mouse-mab/9206?site-search-type=Products&N=4294956287&Ntt=%239206s&fromPage=plp&_requestid=2582991). Additional validation we performed included Western blotting (bands at the expected MW of phoshorylated p70 and p85 S6 kinases and the disappearance upon mTORC1 inhibition by Torin 1 treatment).

Anti-4E-BP1 (Cell Signaling, #9644): according to the manufacturer, this monoclonal antibody detects endogenous levels of total 4E-BP1 protein from human origin; cited in > 1116 publications (https://www.cellsignal.com/products/primary-antibodies/4e-bp1-53h11-rabbit-mab/9644?site-search-type=Products&N=4294956287&Ntt=%239644%29&fromPage=plp&_requestid=2583398).

Anti-p70 S6 Kinase (Cell Signaling, #2708T): according to the manufacturer, this monoclonal antibody detects endogenous levels of total p70 S6 kinase protein. The antibody also recognizes p85 S6 kinase; cited in >1500 publications (https://www.cellsignal.com/products/primary-antibodies/p70-s6-kinase-49d7-rabbit-mab/2708?site-search-type=Products&N=4294956287&Ntt=%232708t%29%3A&fromPage=plp&_requestid=2583783).

Anti-vinculin (Cell Signaling; #13901): according to the manufacturer, this monoclonal antibody recognizes endogenous levels of total vinculin protein. This antibody also reacts with metavinculin, a 145 kDa splice variant of vinculin; cited in >390 publications (https://www.cellsignal.com/products/primary-antibodies/vinculin-e1e9v-xp-rabbit-mab/13901?site-search-type=Products&N=4294956287&Ntt=%2313901%29%3A&fromPage=plp&_requestid=2584007).

Eukaryotic cell lines

Policy information about cell lines and Sex and Gender in Research

Cell line source(s)	The hiPSC HPSI0214i-kucg_2 (male) and HPSI0214i-wibj_2 (female) cell lines were sourced from the HipSci Consortium (https://www.hipsci.org/) through the European Collection of Authenticated Cell Cultures (ECACC). HEK 293T/17 cells were obtained from ATCC (CRL-11268). Lenti-X™ 293T cells were obtained from Takara Bio (#632180).
Authentication	hiPSC, NPC, neurons, and cardiomyocytes were authenticated by the analysis of marker gene expression in RNA-seq datasets and the presence of the respective proteins by fluorescence microscopy. HEK 293T/17 and Lenti-X™ 293T cells were not authenticated.
Mycoplasma contamination	All cell lines used in this study were tested negative for mycoplasma contamination.
Commonly misidentified lines (See ICLAC register)	No commonly misidentified lines were used.

Plants

Seed stocks	*Report on the source of all seed stocks or other plant material used. If applicable, state the seed stock centre and catalogue number. If plant specimens were collected from the field, describe the collection location, date and sampling procedures.*
Novel plant genotypes	*Describe the methods by which all novel plant genotypes were produced. This includes those generated by transgenic approaches, gene editing, chemical/radiation-based mutagenesis and hybridization. For transgenic lines, describe the transformation method, the number of independent lines analyzed and the generation upon which experiments were performed. For gene-edited lines, describe the editor used, the endogenous sequence targeted for editing, the targeting guide RNA sequence (if applicable) and how the editor was applied.*
Authentication	*Describe any authentication procedures for each seed stock used or novel genotype generated. Describe any experiments used to assess the effect of a mutation and, where applicable, how potential secondary effects (e.g. second site T-DNA insertions, mosiacism, off-target gene editing) were examined.*

ChIP-seq

Data deposition

☒ Confirm that both raw and final processed data have been deposited in a public database such as GEO.

☒ Confirm that you have deposited or provided access to graph files (e.g. BED files) for the called peaks.

Data access links
May remain private before publication.

https://www.ncbi.nlm.nih.gov/geo/query/acc.cgi?acc=GSE227928
(reviewer token: mdmzwugkhputxkz)

Files in database submission

Too many files to list, please see GEO accession.

Genome browser session
(e.g. UCSC)

NA

Methodology

Replicates

All ChIP-seq experiments were performed on two biological replicates per cell type examined in this study, with the exception of H3K27me3 ChIP-seq experiments and H3K4me3 ChIP-Seq in NPC, which were performed as single replicates.

Sequencing depth

ChIP-seq library sequencing was performed on an Illumina NovaSeq platform, with 110bp paired-end reads. All libraries had > 30 million reads and 60-76% uniquely mapped reads per library. Full details below:

Sample Reads Uniquely mapped number Uniquely mapped %
WT_RPC1_k_hiPSC_rep1 80,515,257 54,829,218 68.10%
WT_RPC1_k_hiPSC_rep2 83,868,794 57,410,834 68.45%
WT_RPC1_w_hiPSC_rep1 56,632,856 37,844,794 66.82%
WT_RPC1_w_hiPSC_rep2 55,982,708 37,859,040 67.63%
WT_RPC1_k_NPC_rep1 101,693,829 71,746,094 70.55%
WT_RPC1_k_NPC_rep2 81,257,191 57,657,985 70.96%
WT_RPC1_k_neurons_rep1 98,243,649 65,871,592 67.05%
WT_RPC1_k_neurons_rep2 126,844,321 87,688,888 69.13%
WT_RPC1_k_CM_rep1 84,380,879 58,631,896 69.48%
WT_RPC1_k_CM_rep2 77,730,654 55,015,449 70.78%
WT_RPC1_HEK293T_rep1 75,085,959 51,484,833 68.57%
WT_RPC1_HEK293T_rep2 69,435,742 50,403,901 72.59%
WT_BRF1_k_hiPSC_rep1 69,651,303 42,859,623 61.53%
WT_BRF1_k_hiPSC_rep2 65,541,911 47,193,717 72.01%
WT_BRF1_k_NPC_rep1 62,636,853 43,866,556 70.03%
WT_BRF1_k_NPC_rep2 60,150,585 42,949,150 71.40%
WT_BRF1_k_neurons_rep1 61,790,148 43,711,076 70.74%
WT_BRF1_k_neurons_rep2 66,923,872 45,695,831 68.28%
WT_BRF1_k_CM_rep1 56,767,496 38,857,672 68.45%
WT_BRF1_k_CM_rep2 70,591,953 48,701,750 68.99%
WT_H3K4me3_k_hiPSC_rep1 79,476,847 54,218,854 68.22%
WT_H3K4me3_k_hiPSC_rep2 66,452,058 43,025,129 64.75%
WT_H3K4me3_k_NPC_rep1 70,304,132 50,679,818 72.09%
WT_H3K4me3_k_neurons_rep1 87,149,242 61,670,227 70.76%
WT_H3K4me3_k_neurons_rep2 62,893,308 44,984,001 71.52%
WT_H3K4me3_k_CM_rep1 84,122,956 61,107,086 72.64%
WT_H3K4me3_k_CM_rep2 92,281,152 66,120,972 71.65%
WT_H3K27me3_k_hiPSC_rep1 78,287,550 48,793,806 62.33%
WT_H3K27me3_k_NPC_rep1 128,691,861 94,942,665 73.78%
WT_H3K27me3_k_neurons_rep1 86,560,571 59,314,104 68.52%
WT_H3K27me3_k_CM_rep1 100,608,444 75,254,256 74.80%
WT_H3K9me3_k_hiPSC_rep1 74,537,018 54,404,208 72.99%
WT_H3K9me3_k_NPC_rep1 80,831,019 58,753,343 72.69%
WT_H3K9me3_k_neurons_rep1 75,643,034 53,672,033 70.95%
MAF1_ctrl_RPC1_k_hiPSC_rep1 75,815,702 51,798,493 68.32%
MAF1_ctrl_RPC1_k_hiPSC_rep2 72,318,018 50,548,859 69.90%
MAF1_KD_RPC1_k_hiPSC_rep1 72,099,050 52,361,187 72.62%
MAF1_KD_RPC1_k_hiPSC_rep2 86,229,444 60,336,854 69.97%
MAF1_ctrl_RPC1_k_NPC_rep1 86,098,574 58,337,615 67.76%
MAF1_ctrl_RPC1_k_NPC_rep2 31,700,275 22,666,980 71.50%
MAF1_KD_RPC1_k_NPC_rep1 70,440,589 47,449,928 67.36%
MAF1_KD_RPC1_k_NPC_rep2 77,231,700 54,075,167 70.02%
MAF1_ctrl_RPC1_k_NPCderived_rep1 80,812,139 58,324,605 72.17%
MAF1_ctrl_RPC1_k_NPCderived_rep2 111,879,883 78,916,068 70.54%
MAF1_KD_RPC1_k_NPCderived_rep1 96,711,579 66,940,061 69.22%
MAF1_KD_RPC1_k_NPCderived_rep2 142,919,671 100,032,182 69.99%
ProTGG_bodyEdit_RPC1_k_hiPSC_rep1 37,535,143 22,576,642 60.15%
ProTGG_bodyEdit_RPC1_k_hiPSC_rep2 26,027,666 16,797,930 64.54%
ProTGG_bodyEdit_RPC1_k_NPC_rep1 46,713,583 33,263,504 71.21%

ProTGG_bodyEdit_RPC1_k_NPC_rep2 58,655,074 41,317,943 70.44%
ProTGG_upstreamEdit_RPC1_k_hiPSC_rep1 28,084,522 18,838,232 67.08%
ProTGG_upstreamEdit_RPC1_k_hiPSC_rep2 30,599,758 20,104,269 65.70%
ProTGG_upstreamEdit_RPC1_k_NPC_rep1 30,287,901 22,374,858 73.87%
ProTGG_upstreamEdit_RPC1_k_NPC_rep2 34,271,870 24,755,841 72.23%
ProTGG_bodyEdit_BRF1_k_hiPSC_rep1 81,161,914 60,397,498 74.42%
ProTGG_bodyEdit_BRF1_k_hiPSC_rep2 96,259,734 70,116,314 72.84%
ProTGG_bodyEdit_BRF1_k_NPC_rep1 49,297,297 37,661,883 76.40%
ProTGG_bodyEdit_BRF1_k_NPC_rep2 62,869,462 46,707,897 74.29%
ProTGG_upstreamEdit_BRF1_k_hiPSC_rep1 65,353,550 47,632,397 72.88%
ProTGG_upstreamEdit_BRF1_k_hiPSC_rep2 68,648,862 51,492,865 75.01%
ProTGG_upstreamEdit_BRF1_k_NPC_rep1 64,534,453 48,190,454 74.67%
ProTGG_upstreamEdit_BRF1_k_NPC_rep2 44,920,325 28,873,475 64.28%
WT_RPC7a_k_hiPSC_rep1 28,173,190 18,642,449 66.17%
WT_RPC7a_k_hiPSC_rep2 33,137,234 24,040,817 72.55%
POLR3G_ctrl_RPC1_k_hiPSC_rep1 58,112,790 39,861,878 68.59%
POLR3G_ctrl_RPC1_k_hiPSC_rep2 65,067,136 46,919,952 72.11%
POLR3G_KD_RPC1_k_hiPSC_rep1 62,249,136 43,208,968 69.41%
POLR3G_KD_RPC1_k_hiPSC_rep2 57,939,450 42,281,264 72.97%
POLR3G_ctrl_Rapa_RPC1_k_hiPSC_rep1 35,674,600 24,354,478 68.27%
POLR3G_ctrl_Rapa_RPC1_k_hiPSC_rep2 37,339,132 25,634,855 68.65%
POLR3G_KD_Rapa_RPC1_k_hiPSC_rep1 40,067,445 25,751,532 64.27%
POLR3G_KD_Rapa_RPC1_k_hiPSC_rep2 44,752,449 29,952,037 66.93%
w_hiPSC_Input_ChIP 74,182,549 54,018,380 72.82%
NPC_Input_ChIP 91,246,822 69,375,429 76.03%
CM_Input_ChIP 100,695,245 75,124,740 74.61%
HEK293T_Input_ChIP 72,935,763 54,048,987 74.10%

Antibodies

Anti-POLR3A/RPC1 antibody (Cell Signaling Technology, #12825)
Anti-BRF1 (Abcam, #ab264191)
Drosophila spike-in antibody (Active Motif, #61686)
Anti-H3K4me3 (Active Motif, #39159)
Anti-H3K27me3 (Millipore, #07-449)
Anti-H3K9me3 (Cell Signaling, #13969)
Anti-POLR3G/RPC7α (Santa Cruz, #sc21754)

Peak calling parameters

Reads were aligned to the GRCh38 reference genome using STAR with allowing up to one mismatch per read, a maximum of ten alignment positions, end-to-end alignment, prohibited introns, only one alignment reported per read.
Peak calling was performed on duplicate-filtered alignments using MACS callpeak v2.2.6, supplying ChIP input samples from HPSI0214i-kucg_2 for kucg-2 hiPSC and CM datasets, HPSI0214i-wibj_2 for wibj_2 hiPSC datasets, and from HPSI0214i-kucg_2-derived NPC for NPC and neuron datasets with the following parameters:
-g hs --slocal 500 -B --keep-dup all --nomodel --extsize {FRAG} --SPMR
where {FRAG} corresponds to the estimated fragment size from cross-strand correlation analysis. For H3K4me3, H3K27me3 and H3K9me3 the --broad parameter was additionally specified to call broad peaks for these chromatin marks.
Finally, peaks were filtered for blacklist regions as specified by the ENCODE GRCh38 blacklist file (https://www.encodeproject.org/files/ENCFF356LFX/).

Data quality

FastQC was run on all FASTQ files to assess general sequencing quality. Mapping stats were generated automatically by STAR and assessed. For all ChIP-seq samples except H3K27me3, narrow peaks were called with an FDR < 0.05. Called peaks were validated for RPC1, RPC7, and BRF1 by assessing overlaps with predicted Pol III targets, specifically tRNAs, revealing high congruency with previous reports, and near-complete overlap of tRNA peaks between biological replicates. Full details of number of peaks given below:

Sample Peaks
WT_RPC1_k_hiPSC_rep1 1,113
WT_RPC1_k_hiPSC_rep2 979
WT_RPC1_w_hiPSC_rep1 666
WT_RPC1_w_hiPSC_rep2 940
WT_RPC1_k_NPC_rep1 348
WT_RPC1_k_NPC_rep2 340
WT_RPC1_k_neurons_rep1 341
WT_RPC1_k_neurons_rep2 293
WT_RPC1_k_CM_rep1 537
WT_RPC1_k_CM_rep2 542
WT_RPC1_HEK293T_rep1 882
WT_RPC1_HEK293T_rep2 690
WT_BRF1_k_hiPSC_rep1 1,088
WT_BRF1_k_hiPSC_rep2 733
WT_BRF1_k_NPC_rep1 334
WT_BRF1_k_NPC_rep2 326
WT_BRF1_k_neurons_rep1 325
WT_BRF1_k_neurons_rep2 378
WT_BRF1_k_CM_rep1 457
WT_BRF1_k_CM_rep2 511
WT_H3K4me3_k_hiPSC_rep1 25,807
WT_H3K4me3_k_hiPSC_rep2 23,820
WT_H3K4me3_k_NPC_rep1 19,780

WT_H3K4me3_k_neurons_rep1 18,340
WT_H3K4me3_k_neurons_rep2 18,553
WT_H3K4me3_k_CM_rep1 25,575
WT_H3K4me3_k_CM_rep2 24,835
WT_H3K27me3_k_hiPSC_rep1 34,934
WT_H3K27me3_k_NPC_rep1 19,160
WT_H3K27me3_k_neurons_rep1 110,582
WT_H3K27me3_k_CM_rep1 76,439
WT_H3K9me3_k_hiPSC_rep1 455,378
WT_H3K9me3_k_neurons_rep1 342,506
WT_H3K9me3_k_NPC_rep1 377,453
MAF1_ctrl_RPC1_k_hiPSC_rep1 2,063
MAF1_ctrl_RPC1_k_hiPSC_rep2 1,761
MAF1_KD_RPC1_k_hiPSC_rep1 2,148
MAF1_KD_RPC1_k_hiPSC_rep2 2,635
MAF1_ctrl_RPC1_k_NPC_rep1 740
MAF1_ctrl_RPC1_k_NPC_rep2 414
MAF1_KD_RPC1_k_NPC_rep1 1,019
MAF1_KD_RPC1_k_NPC_rep2 884
MAF1_ctrl_RPC1_k_NPCderived_rep1 628
MAF1_ctrl_RPC1_k_NPCderived_rep2 677
MAF1_KD_RPC1_k_NPCderived_rep1 967
MAF1_KD_RPC1_k_NPCderived_rep2 839
ProTGG_bodyEdit_RPC1_k_hiPSC_rep1 1,338
ProTGG_bodyEdit_RPC1_k_hiPSC_rep2 2,180
ProTGG_bodyEdit_RPC1_k_NPC_rep1 282
ProTGG_bodyEdit_RPC1_k_NPC_rep2 356
ProTGG_upstreamEdit_RPC1_k_hiPSC_rep1 893
ProTGG_upstreamEdit_RPC1_k_hiPSC_rep2 999
ProTGG_upstreamEdit_RPC1_k_NPC_rep1 314
ProTGG_upstreamEdit_RPC1_k_NPC_rep2 383
ProTGG_bodyEdit_BRF1_k_hiPSC_rep1 722
ProTGG_bodyEdit_BRF1_k_hiPSC_rep2 681
ProTGG_bodyEdit_BRF1_k_NPC_rep1 253
ProTGG_bodyEdit_BRF1_k_NPC_rep2 123
ProTGG_upstreamEdit_BRF1_k_hiPSC_rep1 658
ProTGG_upstreamEdit_BRF1_k_hiPSC_rep2 742
ProTGG_upstreamEdit_BRF1_k_NPC_rep1 272
ProTGG_upstreamEdit_BRF1_k_NPC_rep2 102
WT_RPC7a_k_hiPSC_rep1 425
WT_RPC7a_k_hiPSC_rep2 175
POLR3G_ctrl_RPC1_k_hiPSC_rep1 906
POLR3G_ctrl_RPC1_k_hiPSC_rep2 737
POLR3G_KD_RPC1_k_hiPSC_rep1 725
POLR3G_KD_RPC1_k_hiPSC_rep2 672
POLR3G_ctrl_Rapa_RPC1_k_hiPSC_rep1 734
POLR3G_ctrl_Rapa_RPC1_k_hiPSC_rep2 842
POLR3G_KD_Rapa_RPC1_k_hiPSC_rep1 650
POLR3G_KD_Rapa_RPC1_k_hiPSC_rep2 719
w_hiPSC_Input_ChIP NA
NPC_Input_ChIP NA
CM_Input_ChIP NA
HEK293T_Input_ChIP NA

Software

Software used to analyze ChIP-seq data includes: STAR, MACS, Trim Galore, Picard Tools, mmquant, deepTools, bedtools, ComplexHeatmap, DiffBind and ChIPpeakAnno. Software versions stated above.
