# Supplementary figures and images for: Selective gene expression maintains human tRNA anticodon pools during differentiation
Source: Nat Cell Biol. 2024 Jan 8;26(1):100–12. doi: 10.1038/s41556-023-01317-3 (PMC10791582; doi:10.1038/s41556-023-01317-3)

Figure 6a

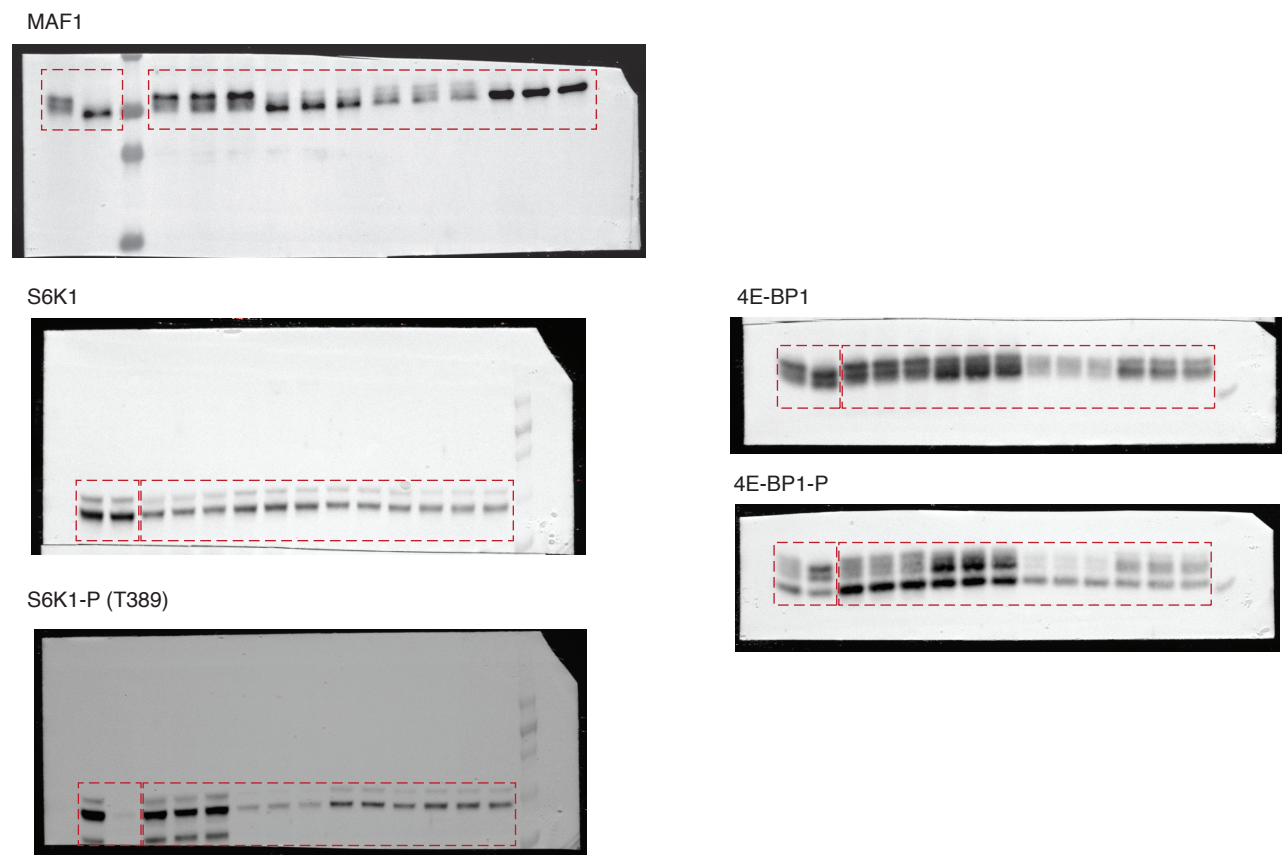

Figure 6b

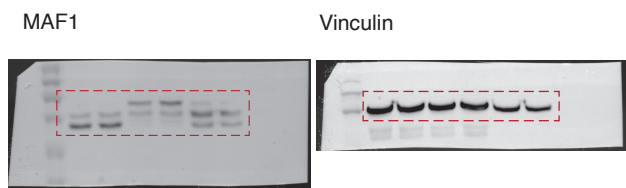

Figure 6c

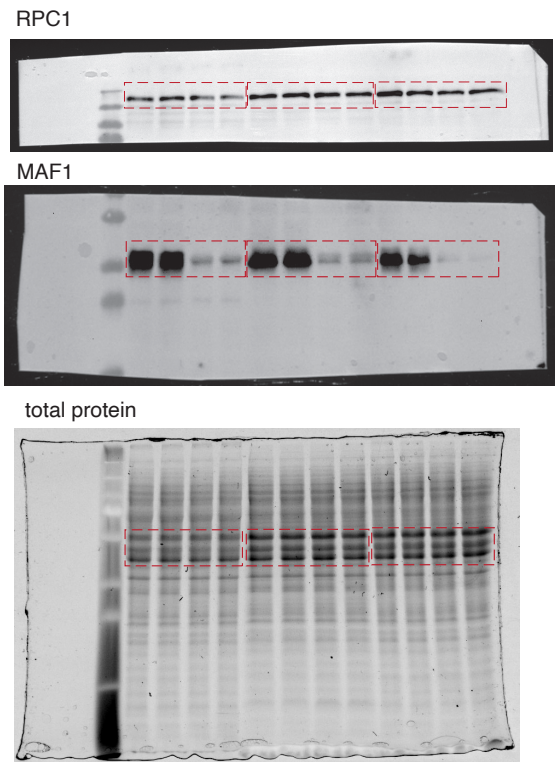

Supplement: Supplementary file 10 — Unprocessed western blots. [file 41556_2023_1317_MOESM10_ESM.pdf]
