# Supplementary material for: Selective gene expression maintains human tRNA anticodon pools during differentiation
Source: Nat Cell Biol. 2024 Jan 8;26(1):100–12. doi: 10.1038/s41556-023-01317-3 (PMC10791582; doi:10.1038/s41556-023-01317-3)

Extended Data Figure 1

tRNA-Asn-GUU-1

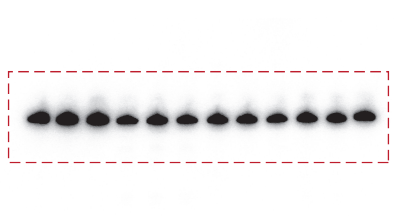

tRNA-Arg-UCU-4

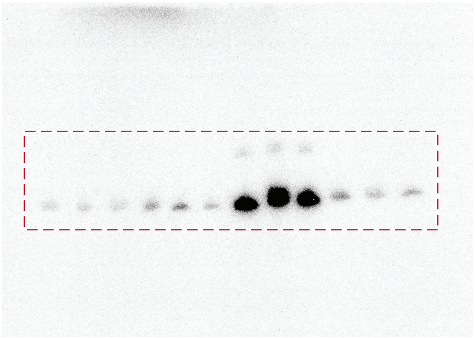

tRNA-Gly-CCC-2

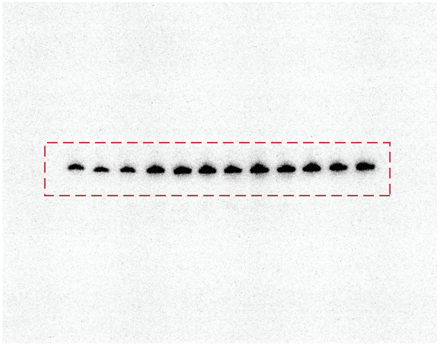

Extended Data Figure 4

RPC1

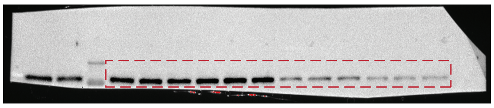

total protein

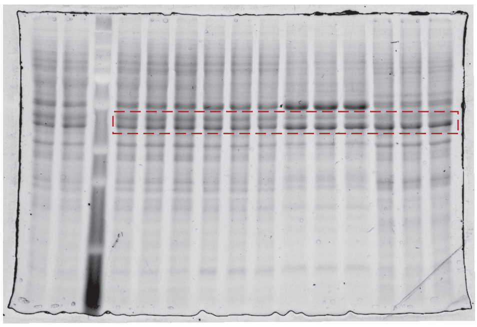

RPC2

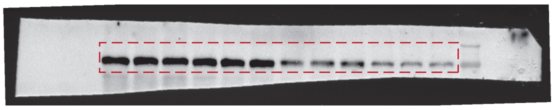

total protein

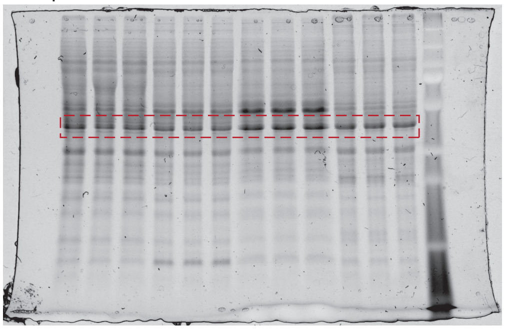

Extended Data Figure 8

RPC7α

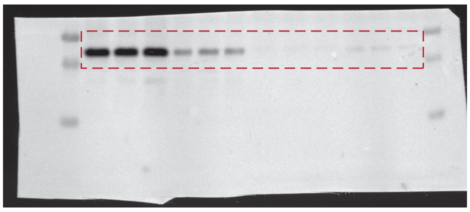

RPC7α

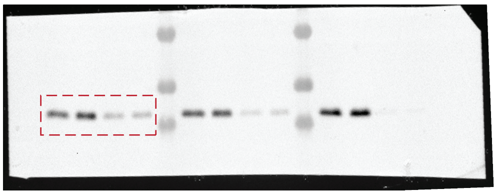

total protein

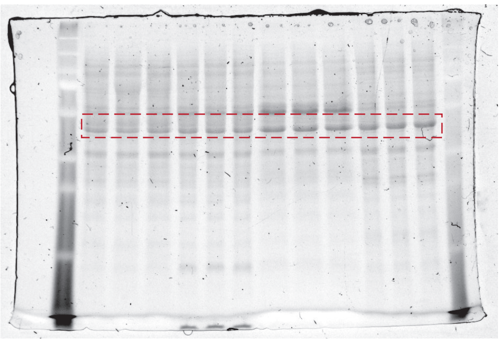

total protein

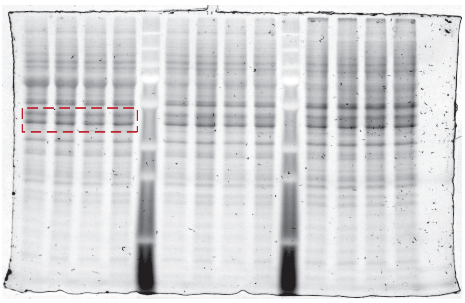

Supplement: Supplementary file 19 — Unprocessed northern and western blots in Extended Data Figs. 1–8. [file 41556_2023_1317_MOESM19_ESM.pdf]
